# PET scanning for evaluation of bone metabolism

**DOI:** 10.3109/17453670903487040

**Published:** 2009-12-04

**Authors:** Jens Sörensen, Gösta Ullmark

**Affiliations:** ^1^Dept. of Nuclear Medicine, Uppsala University Hospital, Uppsala; ^2^Dept. of Orthopedics, Gävle Hospital and Center for Research and Development, Uppsala University/County Council of Gävleborg, Sweden

## Introduction

*Sir*–With great interest we read the paper of Ullmark et al. on using positron emission tomography (PET) to assess bone metabolic activity (Ullmark et al. 2009). PET is an imaging tool that can provide functional rather than morphological information, and has thus attracted a lot of attention. The prerequisites for proper analysis and subsequent sufficient interpretation are very important, and most of these originate from the principles of PET. Unfortunately, Ullmark et al. do not address these important issues and, to avoid misinterpretations, we wish to highlight some of these important points.

The principle of positron emission tomography PET scanning is based on a short-lived radioactive tracer, which decays by emitting a positron. The tracers can exist as single isotopes or be incorporated in larger molecules, depending on the application. This method suffers from low spatial resolution. Apart from single-positron emission tomography (SPECT), all PET systems utilize coincidence detection of the annihilation photons from positron decay. Since the paired gamma rays from the annihilation of the positron are anti-parallel, the detection of the gamma rays determines a line of response (LOR) along which the annihilation took place. Resolution is thus determined by the size/density of the detectors that are most often placed in a ring around the subject. However, the LOR is not completely linear and the gamma rays are released in a direction of 180° ± 0.5°. This physical phenomenon further limits the resolution. The spatial resolution of most clinical PET scanners is therefore only about 6–8 mm (Townsend 2004). Hence, the resulting images are known to be affected by partial-volume effects, which can cause small regions with high tracer uptake to be imaged as having an artificially low concentration and vice versa (Soret et al. 2007). When comparing regions of interest (ROIs), it is therefore important for the accuracy that the size is sufficiently high or that the tissue surrounding the ROIs has a comparable uptake of the tracer. For the reader to be able to interpret PET data, describing the size of the ROI is as important as the obvious adding of standard deviations to the results. The three basic analytical methods with or without blood sampling will not be addressed here.

In orthopedic approaches, the use of the fluoride isotope [^18^F] is of particular interest because of its incorporation into the bone crystals. However, due to the rate of bone formation, [^18^F] is not incorporated into bone during a 1- to 2-hour scan. The fluoride ion exchanges with the hydroxyl groups in the hydroxyapatite crystal of bone (Ca_10_(PO_4_)_6_OH_2_) to form fluoroapatite (Ca_10_(PO_4_)_6_F_2_), and this can be interpreted as a 4-step event, which was originally described by Blau et al. as early as 1962—long before PET gained its clinical popularity and accessibility. The first 2 steps from the blood through extracellular space to the shell of bound water around exposed crystals are very rapid (minutes), step 2 being the irreversible step. Step 3 is the traveling of the tracer onto the crystal surface, and this is probably measured in hours. The final step of incorporation, step 4, may take days or weeks. Consequently, areas of high uptake result from processes that increase the exposed bone crystal surface and/or increase blood flow. Thus, areas of osteoclastic and osteoblatic activity are measured equally, and this is of crucial importance when interpreting these data sets (Blau et al. 1972).

By directly translating SUV to “bone forming activity”, Ullmark et al. draw a dicey conclusion. This translation is apparently based on a study by Piert et al. correlating metabolic activity in the vertebral body from PET and histomorphometry in an iliac crest biopsy (Piert et al. 2001). However, that study investigated bone blood flow and metabolic rate in untreated bone, which most likely presents a steady state metabolic rate. This is obviously very different from new bone formation in an allograft-implanted area. The findings at 1 week postoperatively in Ullmark's study could very well be due to impaired blood flow in the impacted graft rather than being an assessment of metabolic rate per se (Berding et al. 1995, Piert et al. 1998). Furthermore, there is evidence that radiotracer uptake is not dependent on osteoblast number, but concentration of bone-forming minerals (Toegel et al. 2006). It is therefore important to notice that areas of osteoclastic and osteoblastic activity are labeled equally, and thus increased resorption would also result in increased tracer uptake without it necessarily resulting in a net increase in bone volume (Blau et al. 1972, Genant et al. 1974).

Combined PET/CT and (most recently) PET/MRI scanners make it possible to correlate the functional data from PET with morphological images (Foldager et al. 2008). This has two advantages. First, it gives an exact morphological location of the metabolic event and secondly, ROIs can be made from CT or MRI images alone, which makes the placement of these more accurate and furthermore limits possible inter-observer errors.

PET is indeed a fast-growing modality and both PET and cyclotron centers are opening and expanding in the western countries, thereby increasing accessibility for researchers in the field of orthopedics. We therefore find it very important that the limitations as well as the opportunities of this powerful tool are properly addressed and well understood to avoid misinterpretations and misunderstanding of this rather complex imaging modality.

## Casper Bindzus Foldager, Michael Bendtsen, Cody Bünger

Orthopaedic Research Laboratory,

Aarhus University Hospital, Denmark

foldager@ki.au.dk

*Sir*—We welcome this discussion initiated by Foldager et al. for several reasons. Fluoride PET has been available for 10–15 years in academic PET centers such as Uppsala and Aarhus, but reports on the use of this tool for orthopedic research are still scarce. A large increase in availability of fluoride PET is anticipated in the coming years, as more clinically-oriented PET centers introduce fluoride PET/CT for routine scanning in benign and malignant bone diseases (Grant et al. 2008). This development is good news for researchers in orthopedics, and will probably spark a range of novel approaches for non-invasive studies of bone metabolism. Nonetheless, each new technology comes with its pros and cons.

The true power of PET lies in its capacity for absolute quantification, i.e. the concentration of tracer substance is measured with high accuracy. When novel tracers and applications are probed for biological relevance, such measurements typically require advanced set-ups and lengthy investigations. Foldager et al. reference their own paper (2008) in which the 18F-fluoride flux from plasma to bone was calculated using simultaneous radioactivity sampling of tissue, by dynamic imaging, and blood, by arterial lines. However, to facilitate a human study in a clinical setting with repeated measurements, it appears highly relevant to search for a simplified approach. In a previous paper (Sörensen et al. 2003) we validated the use of SUV measurements in human femoral allografts 40 min after tracer injection against the more rigorous method also employed by Foldager et al. The correlation over a wide range of metabolic activity was r = 0.97, indicating that SUVs and Gjedde-Patlak plots perform equally in this setting. Furthermore, the long-term reproducibility of both measurements is 12–14% in normal bone (Frost et al. 2008). The use of SUVs for quantification also allows the entire skeleton to be examined in one session. That first paper also discussed the PET technology in general terms. We are confident about the use of simplified quantification in the hip area, but would encourage future researchers engaging other parts of the skeleton to perform validating work.

Another important technical aspect to take into consideration when planning a study is the details related to the graphical analysis of images. Foldager et al. specifically point out the partial volume problem (i.e. the measurement of local activity is affected by the activity in surrounding tissues due to blurring in images). This is a fact that has to be dealt with in all imaging studies to some degree, and the rationale for our use of ROIs of at least 1 cm^2^.

### Bone mineralization

Foldager et al. raise an issue on the interpretation of fluoride uptake and refer to the original work performed by Blau et al. (1972). The question is whether or not the PET signal is exclusively related to new bone formation. It was previously assumed that 18F-fluoride would accumulate in mineral created both by osteoblasts and clasts. More recent information appears to support a view that 18F-fluoride is bound only to the mineral formed and fixated by the osteoblasts (Anderson 2003, Toegel et al. 2006). Osteoclasts degrade bone material internally and expel the debris by a mechanism of transcytosis to the extracellular space (Salo et al. 1997). Although not rigorously proven, naked mineral in resorption zones to which free fluoride ions from the extracellular space could attach is not likely to exist in vivo. For example, osteolytic metastases only have a faint rim uptake on PET and radiated bone has virtually no uptake at all despite continuous demineralization and relatively normal perfusion. This means that an integrated assessment of bone remodeling cannot be performed with fluoride PET alone.

### The problem of PET and CT

As Foldager et al. state, the fusion of images from both PET and either MRI or CT greatly improves our understanding of the biology. So far, PET/MRI exists only as prototypes. Integrated PET/CT devices have been commercially available for a few years. The first clinical PET/CT scanner in Sweden was installed in early 2005 in Uppsala. Some of the projects in our group started earlier than that. For the experienced interpreter, uptake in fluoride PET images is relatively easy to locate anatomically even without CT. The use of the integrated device poses a problem in orthopedics, because of the artifacts produced by CT at the level of metallic implants. Since the CT component of PET/CT is used to correct for photon attenuation, the final PET images also contain artifacts (Goerres et al. 2003). This device is therefore not very useful in several of our projects. Older-generation PET scanners correctly assess the uptake concentration surrounding implants and are therefore preferable for quantification. Fortunately, the PET Center in Uppsala has 2 of these machines. In some ongoing projects, we use separately obtained CT or MRI for more exact anatomical location. Still, patient motion during a scan or incorrect positioning of fused images must be taken into account.

To summarize, we agree with Foldager et al. that there are several technical aspects of PET that need to be attended to before meaningful results can be obtained. Most likely, very few orthopedics researchers will opt for mastering this highly technical discipline and successful projects therefore require early liaisons between specialists.
